# Psychometrics of Drawmetrics: An Expressive–Semantic Framework for Personality Assessment

**DOI:** 10.3390/bs16010135

**Published:** 2026-01-17

**Authors:** Larry R. Price

**Affiliations:** 1 Biostatistics and Psychometrics, Joe & Teresa Long School of Medicine, The University of Texas Health Science Center at San Antonio, San Antonio, TX 78229, USA; lrprice_phd@outlook.com; 2 AttituX Ltd., San Marcos, TX 78666, USA

**Keywords:** Drawmetrics, Five-Factor Model, semantic linking, trait mapping, sentence transformers, network psychometrics

## Abstract

This study examines whether Drawmetrics (DM), an expressive–semantic personality system, can be linked with the Five-Factor Model (Big Five) through an embedding-based mapping approach and network psychometric methods. A total of 185 participants completed both the DM assessment and the IPIP-NEO 120 Big Five inventory. DM term outputs were embedded using a miniLM sentence-transformer and aggregated into 30 facet composites, with six composites per domain. Big Five facet composites were extracted from standardized reports and harmonized to canonical facet names. Analyses focused on the overlap sample (N = 148) with valid scores on both instruments. DM composites demonstrated strong internal structure and high stability indices. Substantial semantic-space alignment was observed between DM term language and Big Five facet language, supporting interpretable linking. However, person-level correlations between DM and Big Five domains were modest (mean |r| ≈ 0.07; Spearman similar), with the largest facet-level association at |r| ≈ 0.26. DM appears to represent a coherent expressive–semantic trait space that is related to, but not isomorphic with, Big Five traits. These findings support a linking rather than equivalence interpretation and highlight the need for future research on scaling, reliability, range restriction, and criterion validation.

## 1. Introduction

The present study is framed as a proof-of-concept evaluation of an expressive–semantic measurement framework. Accordingly, the goal is not population-level validation, but to demonstrate the feasibility, internal coherence, and construct-aligned behavior of measures produced via anchor-based semantic linking.

### 1.1. Background and Problem

Personality evaluation is fundamental to selection, development, and applied decision-making in organizational and clinical settings. The Five-Factor Model (Big Five) has long served as the primary lexical framework for describing broad personality trait domains (John & Srivastava, 1999). However, this model relies on self-report ratings and language-based item responses. In contrast, Drawmetrics (DM) assesses personality by eliciting expressive outputs, specifically brief terms associated with standardized drawings. Computational semantics are then used to map these outputs into a structured trait space. This paper investigates whether DM can be meaningfully linked to the Big Five by (a) evaluating the internal structure and stability of DM composites, (b) quantifying the semantic-space alignment between DM term language and Big Five facet language (structural homology), and (c) estimating person-level convergence between DM and Big Five scores within the same cohort. Alignment is interpreted as evidence for translation or linking, rather than as proof of score equivalence or interchangeability.

### 1.2. Conceptual Gap

To improve an organization’s success, it needs specific personality-based behavioral data that shows how employees work together, stay practical, and generate new ideas. The geometric structure of personality can be revealed through the analysis of linguistic and expressive data that provides rich information for psychometric analyses. The IPIP-NEO-120 (Costa & McCrae, 1992) assesses how people describe themselves and others using verbal, self-report-based language. DM uses nonverbal and semantic production to understand personality structure, as people express their personality through their expressive–semantic manifestations in doodling and term association. DM employs personality assessment through expressive methods that function independently of language needs, introspection, and response bias. The DM approach to personality assessment has yet to be rigorously evaluated psychometrically. This study addresses this gap in research literature.

### 1.3. Significance of the Study

Lexical terms were encoded using a fixed, pre-specified Sentence-Transformers embedding model (all-MiniLM-L6-v2), which projects text into a shared semantic vector space. Further implementation details are not discussed, as they do not directly pertain to the linking structure.

## 2. Materials and Methods

### 2.1. Participants

The analytic samples comprised 185 recruited participants. Drawmetrics scoring was available for 176 individuals, and Big Five report data were available for 149 individuals. The overlap sample used for person-level linking analyses included 148 participants. Unless otherwise specified, domain reliability and indicator-level diagnostics were conducted using the Drawmetrics sample (N = 176), while linking analyses utilized the overlap sample (N = 148).

A non-random convenience sampling approach was employed by an official at AttituX Ltd. Pte., who contacted organizational representatives via email to recruit volunteers for a study evaluating the psychometric properties of Drawmetrics (DM) and benchmarking it against a Big Five inventory. The final sample comprised 185 individuals who completed both the DM and Big Five assessments. Big Five results were provided as standardized reports (PDF/TXT), and DM scores were generated using an embedding-based scoring pipeline; thus, the analytic sample was determined by successful scoring and quality control. Usable DM facet composites were available for 176 individuals, and usable Big Five facet composites for 149 individuals. After excluding one known dummy/test form (AT0001) and four non-English Big Five reports (AT0015, AT0074, AT0099, AT0129), the overlap sample for DM and Big Five linking analyses consisted of 148 individuals. All participants received anonymous reports of their DM and Big Five results. Respondent information remained anonymous to the researchers and all study personnel. Informed consent was obtained before assessment administration. No demographic information or personal identifiers were collected to protect participant confidentiality. The sample included individuals from universities, workforce and staffing companies, multinational businesses, and employees at various organizational levels, including C-suite, middle management, and entry-level staff. Geographic representation included the Asia Pacific region (10 countries), Australia and New Zealand, Western Europe (Italy, Spain, France, and Germany), the Middle East (Saudi Arabia and the UAE), and the Americas (Canada).

### 2.2. Measures

DM is a nonverbal assessment that uses expressive-semantic methods to evaluate personality traits by translating symbolic doodles into geometric embeddings. These expressive elements are subsequently analyzed using natural language embeddings, resulting in structured, term-based personality profiles. The DM framework integrates conceptual components from representational measurement theory (RMT) as outlined by Suppes et al. (1990). For instance, Krantz et al. (1971) established empirical relational structures through the expressive connections between semantic and graphical terms. The semantic embedding process transforms these relationships into a vector space, thereby preserving the structural relationships among psychological constructs through a structure-preserving mapping. This study evaluates the extent to which the mapping system maintains the established organizational structure of personality domains using validity assessments. The analyses examine the contribution of representational mapping to relational alignment and assess whether semantic representation preserves the *pattern* of similarities among constructs, without implying equivalence at the score level. This pragmatic measurement approach facilitates the transformation of expressive semantic meanings into geometric representations, as described by Hand (2004) and by Christensen and Golino (2021) in the context of network psychometrics.

When taking the DM assessment, respondents are presented with a set of figures (i.e., stimuli presented as standardized drawings). Respondents use sketches or doodles to create images from the standardized line drawings, which they then assign descriptive terms. Each figure is conceptualized as an “item” or “stimulus,” and each participant creates a doodle for ten separate line drawings, which serve as the basis for the doodling process. For each figure created, participants supply a word or phrase for their doodle that represents attributes describing either a professional orientation or a personal orientation. The object receives its name through a process that connects it to attitudinal terms established by professionals and academics who have served, or who currently serve, as subject-matter experts. Post testing, a person’s data comprises 10 professional terms and 10 personal terms from the doodle drawings. The test contains five foil figures that show neutral or ambiguous content to detect intentional response manipulation through their semantic variations. The system produces individual participant reports that display their personalized attitude terms, their affective-task attitude relationships, and their organizational role suitability. The test duration depends on doodling speed, and most participants complete it within 15 to 20 min. The creation of summary reports proceeds by processing responses and generating an attribute- or term-based inventory organized into personal and professional categories. Automated reports are immediately provided to the person assessed. The IPIP-NEO-120 is a self-report assessment tool that measures the Five-Factor Model of personality by evaluating Openness to Experience, Conscientiousness, Extraversion, Agreeableness, and Neuroticism (Kwapil, 2002). The IPIP-NEO-120 inventory contains 44 short statements that respondents rate on a scale from 1 (strongly disagree) to 5 (strongly agree). The International Personality Pool (IPIP-NEO 120, Johnson, 2014; Goldberg et al., 2006) contains 120 items organized into five domains, with six facets per domain and four items per facet, for a total of 30 facets. The system provides domain-level and facet-level trait scores for assessment. The BFI lacks foil items, but some questions appear in reverse order to reduce acquiescence bias. The system produces reports through domain- and facet-score calculations and normative sample-based standardization. The final output provides T-scores and percentile ranks for each of the five domains, together with their respective facets. The IPIP-NEO 120 inventory requires 20 to 40 min to complete. The system produces reports instantly when it uses automated scoring keys. The five domain scales demonstrate adequate to high internal consistency reliability, with alpha values ranging from 0.79 to 0.86, while all facet scales achieve reliability coefficients above 0.60 (Johnson, 2014). The IPIP-NEO 120 was used in this study.

The Big Five model relies on latent-variable confirmatory factor models, which can obscure structural interdependencies among traits, facets, and behavioral indicators. In contrast, network psychometrics addresses these interdependencies by employing a systems-based approach. Within this framework, observed variables represent underlying traits and states, and network models depict these variables as nodes connected by relationships (Epskamp et al., 2017). This approach elucidates how personality traits form cohesive groups that bridge different domains and occupy either central or peripheral positions within the network. Network models further allow researchers to examine patterns of stability and change over time and to test for structural invariance within networks. The properties derived from network models enhance understanding of both the stability of personality traits and the prediction of future changes. For instance, highly central traits may function as “hubs” (Costantini et al., 2019; Barabási & Albert, 1999), influencing a broad spectrum of behaviors, whereas peripheral traits may fluctuate without destabilizing the overall system. The DM assessment applies semantic term similarity and clustering to link individual responses to personality-related terms, providing detailed insights into how individuals express their core characteristics. Semantic natural language processing (NLP) methods are utilized to analyze DM by measuring structural properties, comparing Big Five domains and facets, and identifying novel traits that traditional assessments may overlook. The Big Five thus serves as a foundation for developing an integrated methodology that combines established trait terminology with modern network analysis and computational text processing techniques.

### 2.3. Data Processing and Quality Control Check

Data processing occurred after importing respondents’ data in Portable Document Format (PDF) and batch-extracting terms using Python (Python Software Foundation, 2023, version 3.11) scripts. Terms were captured then reviewed for accuracy using established DM criteria and, where necessary, corrected using a custom sanitizer to remove duplicates and noise (e.g., “nan,” advertising text, etc.). IPIP-NEO 120 responses were provided as portable document format (PDF), and domain and facet composites were batch-extracted using Python (Python Software Foundation, 2023, version 3.11) scripts written by the author. To prepare the raw data for semantic network analysis, a Python script was written to extract and clean the data (Van Rossum et al., 2009). The script extracts facet terms from DM PDFs and facet/score values from Big Five PDFs, then outputs comma-separated value (CSV) files in both wide and long formats.

A quality control (QC) analysis was subsequently conducted. Among N = 176 participants, the DM pipeline extracted 2197 total term occurrences (mean = 12.55 per participant, SD = 4.55). Following QC filtering, 1921 unique term occurrences were retained for scoring based on presence or absence per anchor. Of the total, 12.6% were removed as within-person duplicates, defined as repeated occurrences of the same anchor term beyond the first. Non-English and noise rates were not applicable, as the file contained counts over a fixed English anchor vocabulary rather than raw tokens. The overall retention rate was 87.4%.

**Figure 1:**
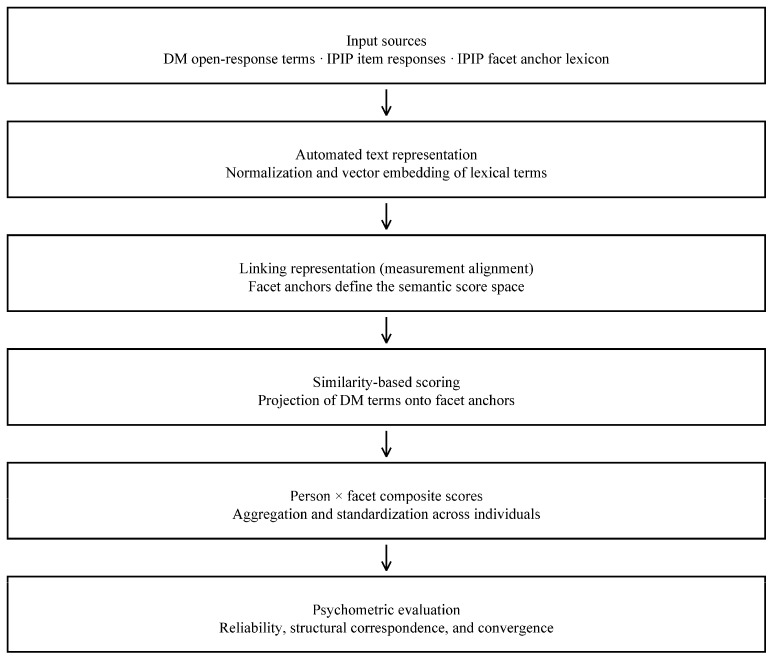
Semantic linking and scoring pipeline for Drawmetrics. Open-response lexical descriptors are represented in a shared semantic space and linked to externally defined facet anchors that specify the measurement frame. Similarity-based projections yield person-by-facet composite scores, which are subsequently standardized and evaluated using established psychometric criteria. The figure emphasizes the conceptual structure of scale linking and score construction rather than algorithmic implementation details.

The input data included open-response lexical descriptors generated from Drawmetrics tasks, item-level responses from the IPIP inventory, and an externally specified IPIP facet anchor lexicon that defined the target measurement frame. Lexical terms were normalized and represented in a shared semantic space using an automated text representation pipeline. Measurement alignment was accomplished by establishing the semantic score space through facet anchors, onto which Drawmetrics terms were projected using similarity-based scoring. Person-by-facet composite scores were calculated by aggregating term similarities and then standardized across individuals. These standardized composites formed the basis for all subsequent psychometric evaluations. Figure 1 provides an overview of the semantic linking and score construction pipeline.

Lexical terms were represented using a fixed, pre-specified Sentence-Transformers embedding model (all-MiniLM-L6-v2), which maps text to a shared semantic vector space. This representation step functions as a stable mapping used for score construction rather than a statistical model estimated from the present sample. Implementation details beyond this representation step are not central to the linking structure and are therefore not elaborated here.

### 2.4. Embedding-to-Score Transformation (Term Embeddings to Standardized DM Composites)

To convert open-response DM term outputs into analyzable psychometric variables, we implemented an embedding-to-score transformation that maps respondent-generated terms into a common trait-language score space aligned to IPIP-NEO facets. First, all respondent DM terms extracted from the PDFs were standardized (e.g., lowercase, trim whitespace, and deduplicate within respondent) to minimize superficial token variation. Each DM term was then embedded using a sentence-transformer model, yielding a fixed-dimensional semantic vector representation for each term $e\left(t\right)\in {\mathbb{R}}^{d}$. In parallel, each IPIP-NEO facet was represented by a predefined facet “anchor” lexicon (facet labels and associated facet-language descriptors), and all anchors were embedded using the same sentence-transformer model to ensure a shared geometric space.

For each respondent term t and each facet f, we computed cosine similarity between the term vector and the facet’s embedded anchor set to quantify semantic proximity of the term to the facet language. Let $A_{f}$ denote the anchor set for facet f; we define the term–facet similarity as

$$s\left(t,f\right)=\frac{1}{|A_{f}|}\underset{a\in A_{f}}{\sum }\cos\left(e\left(t\right),e\left(a\right)\right),$$

with cosine similarity bounded in $\left[-1,\;1\right]$. Respondent-level DM facet composites were obtained by aggregating term–facet similarities across all terms produced by respondent i:

$$x_{if}={\mathrm{mean}}_{t\in T_{i}}s\left(t,f\right),$$

where $T_{i}$ is respondent i’s set of DM terms after preprocessing. This yields a respondent-by-facet matrix $X=\left[x_{if}\right]$ in which each entry summarizes the semantic alignment of a respondent’s DM term set to the language of facet f.

To place DM composites on a common metric appropriate for classical psychometric analyses, each facet composite was standardized across respondents:

$$z_{if}=\frac{x_{if}-{\overset{ˉ}{x}}_{f}}{s_{f}},$$

where the sample mean and standard deviation for each facet are denoted accordingly. Subsequent analyses, including reliability estimation, factor and exploratory graph analysis (EGA), canonical correlation analysis (CCA), and network modeling, were conducted on these standardized DM facet composites and, when applicable, on similarly standardized IPIP-NEO facet composites. Notably, because the facet-anchor lexicon defines the score space, this transformation enables aligning DM outputs with established trait-language constructs. It does not represent an inductive recovery of trait structure from raw DM term endorsement patterns.

### 2.5. Rater Agreement Analysis

Prior to network psychometric analysis, a rating-scale analysis was performed by three subject matter experts using a content rating worksheet to examine the absolute agreement of terms using syntactic and semantic rules on DM and the Big Five. Expert ratings of trait intensity served as a human check of content validity regarding the alignment produced by natural language processing. The content rating worksheet included an ordinal graded response numerical format for sematic and syntactic terminology with numerical values ranging from 1 (no match-terms are entirely unrelated), 2 (weak match-very little similarity, vague or indirect connection), 3 (moderate match-clear similarity in meaning or structure, but notable differences remain), 4 (substantial match-high degree of similarity, terms are closely aligned with minor difference), 5 (exact match-terms are identical or interchangeable in context). Rater reliability was assessed using the intraclass correlation coefficient (ICC; Shrout & Fleiss, 1979) based on a two-way random-effects model for absolute agreement. The intraclass correlation coefficient (ICC = 0.88) revealed high absolute agreement among raters. Established guidelines suggest that ICC values above 0.75 indicate good reliability and those above 0.90 indicate excellent reliability (Shrout & Fleiss, 1979; Koo & Li, 2016).

### 2.6. Data and Network Analysis

Network analyses were conducted to evaluate the reliability, structural, and convergent validity of DM relative to the Big Five. Reliability and validity analyses were performed using conventional and network-based psychometric techniques.

Term scores used for network estimation were computed as defined in the semantic linking and score construction pipeline (Figure 1; Section 2.3 and Section 2.4).

Network construction for both Drawmetrics (DM) and Big Five (B5) treated nodes as descriptor terms, with edges defined as associations among term scores across participants. We estimated sparse partial correlation networks using EBICglasso applied to a Spearman correlation matrix (rank-based to reduce sensitivity to zero inflation and non-normality). We then evaluated edge and centrality stability via bootstrap resampling (1000 replications) and extracted communities using the Louvain clustering method.

### 2.7. Reliability Analysis

Reliability was estimated using established psychometric metrics and new semantic–geometric methods to assess internal consistency and structural stability. The use of a semantic-geometric approach enabled the generation of vectors, which in turn allowed the creation of a correlation matrix. The inter-item (term) correlation matrix was used to estimate domain- and facet-level reliability indices using McDonald’s ω (total). The stability and bias of estimated values were assessed using 95% percentile bootstrap confidence intervals, based on 1000 resampling iterations.

Next, a Schmid–Leiman (bifactor-style) decomposition was used to obtain ω and ω_h_, and to test the hierarchical structure of a general factor and five group factors, each with at least 5 indicators. The assessment of internal semantic reliability used vector-based similarity metrics derived from BERT embeddings. The DM term vectors from each participant were combined to generate similarity matrices, which were used for calculating mean inter-vector coherence as a measure of semantic space consistency. In all, the three methods for evaluating reliability were: classical internal consistency assessment, Schmid–Leiman (bifactor-style) decomposition, and semantic–network stability analysis. Each participant’s DM term vectors were aggregated to compute pairwise cosine similarity matrices, from which mean inter-vector coherence was calculated as an estimate of internal consistency in semantic space. The network’s structural stability was assessed using a bootstrapped structure-recovery approach. Christensen et al. (2020) demonstrated that network recovery frequencies above 0.66 indicate reliable dimensional recovery. Network reliability indices in this study achieved parameter recovery indices exceeding 0.66. Reliability was evaluated using three methods: classical internal consistency, bifactor modeling, and semantic–network stability analysis. The R programming environment (R Core Team, 2025) served as the platform for performing all reliability assessments.

## 3. Results

We report two complementary forms of validity evidence aligned with the measurement pipeline in Figure 1: (a) evidence about the stability of the constructed Drawmetrics composites and their internal coherence, and (b) evidence about person-level linking between Drawmetrics composites and Big Five trait metrics. These address distinct measurement questions and are reported separately.

### 3.1. Composite Score Reliability (Omega-Based Internal Consistency)

Internal consistency and general factor strength for each DM domain were assessed using McDonald’s ω, and Hierarchical ω_h_ (Zinbarg et al., 2005). Reliability and variance partitioning were evaluated using McDonald’s ω and ω_h_ via a Schmid–Leiman decomposition of the indicator correlation matrix; these indices are used here for reliability assessment rather than confirmatory model fit, so SEM fit indices are not reported. These estimates of relational structure stability (internal consistency) appear in Table 1.

Across domains, composite scores showed good to excellent internal consistency (ω = 0.71–0.94; Table 1). Omega hierarchical values indicated that a substantial portion of reliable variance reflects a common component across indicators within domains (ω_h_ = 0.46–0.74 for domains with adequate k), alongside domain-specific variance. Openness showed acceptable ω with relatively higher ω_h_, consistent with heterogeneous indicators that nonetheless share a common component. For Neuroticism (k = 3), ω was high but ω_h_ was not reported because hierarchical partitioning is unstable for very small indicator sets. Bootstrap confidence intervals around ω were narrow, indicating stable estimates in this sample.

### 3.2. Indicator-Level Diagnostics

Indicator-level diagnostics were examined within each Drawmetrics domain using corrected indicator–total correlations (r_it_; Table 2). Values ranged from 0.07 to 0.39 across domains, with mean r_it_ values of 0.20 to 0.24, indicating moderate internal homogeneity for semantically scored, open-response indicators. Sensitivity checks showed that omitting any single indicator produced only trivial changes in ω (Δω < 0.02), consistent with the domain-level composite stability reported in Table 1. Indicator–total correlation summaries are reported in Table 2.

### 3.3. Network Stability and Replicability Using Bootstrap Diagnostics

Network stability was evaluated using bootstrap-based resampling to assess the replicability of the estimated relational organization among semantically scored descriptor terms. Networks were constructed with descriptor terms as nodes and edges representing associations among term scores across participants.

Term scores were computed as defined in the semantic linking and scoring pipeline (Figure 1), yielding a participant × term matrix. For direct comparison, the Big Five network was constructed from Big Five descriptor terms scored using the same semantic representation and scoring procedure, rather than from questionnaire facet composites. Edges were estimated using Spearman rank correlations and EBICglasso partial correlations. Centrality stability coefficients (CS) were estimated using a case-dropping bootstrap, while an edge-weight bootstrap evaluated the consistency of edge estimates across 1000 replications. Community structure was analyzed using Louvain clustering to determine whether the expected five-domain organization emerged.

Bootstrap diagnostics indicated that network features were replicable under resampling. Strength centrality showed excellent stability for Drawmetrics (CS_strength = 0.73), with betweenness and closeness showing good stability (CS = 0.58 and 0.55), consistent with the known sensitivity of these indices. The comparison network showed similar stability (Table 3). Edge-weight bootstrapping supported consistency of edge patterns across resamples (mean edge retention = 0.82 for Drawmetrics; median bootstrapped edge-weight correlation r = 0.88). Community detection recovered an organization consistent with the expected five-domain structure (Q_DM = 0.42), and global connectivity metrics were comparable across networks (global strength 12.4 vs. 13.1; density 0.31–0.33).

### 3.4. Validity Analyses

Validity analyses were designed to address a linking question rather than a strict equivalence claim. We asked: (i) does DM provide evidence of interpretable structure at the indicator and network levels; (ii) does the semantic space induced by DM term language align with the language structure of Big Five facets (structural homology); and (iii) how strongly do DM and Big Five scores covary at the person level in this sample. We report both semantic-space alignment indices (representational similarity) and person-level score correlations, treating the former as evidence for interpretable translation/linking and the latter as evidence about convergence of measured individual differences.

#### 3.4.1. Convergent and Discriminant Validity

Person-level convergent and discriminant evidence was assessed by examining correlations between DM domain composites, calculated as the means of six DM facets per domain, and Big Five domain composites, defined as the means of six canonical IPIP-NEO facets per domain. In the overlap sample (N = 148), the Big Five–DM domain correlation matrix indicated consistently modest associations. Matched-domain Pearson correlations ranged from −0.083 to 0.122 (mean |r| = 0.067), and Spearman correlations were similar (matched-domain ρ ranged from −0.122 to 0.117; mean |ρ| ≈ 0.067). Off-diagonal correlations were of comparable magnitude, indicating limited convergence at the person level within this cohort. At the facet level, the strongest observed association was |r| = 0.259 (pairwise N = 133; Conscientiousness–Orderliness × DM Tends toward sadness). These findings support a “linked-but-not-identical” interpretation: DM and Big Five domains exhibit some overlap, but DM scores in the current scoring scheme do not function as a direct proxy for Big Five trait levels. Observed correlations may be reduced due to range restriction and measurement or temporal instability; Spearman results are reported as a monotone sensitivity check. These indices are therefore interpreted as evidence for representational level linking, while Section 3.4.1 addresses the convergence of individual differences at the person level.

#### 3.4.2. Structural Homology in Representational Space

Representational-space computations use the fixed semantic representations defined in Section 2.3 and Section 2.4. Next, we evaluated whether the relational organization of facets was similar across systems by constructing facet–facet similarity structures and analyzing them with Taxonomic Graph Analysis (TGA), Louvain community detection, and related network indices. In both systems, these analyses identified a coherent five-domain modular organization. Importantly, these results address structural homology (shared organization of trait space) rather than equivalence of person scores; because both systems are represented using English facet descriptors embedded in the same pretrained language model, high configuration-level alignment is expected and is best interpreted as supporting translation/linking between facet vocabularies—not interchangeability of DM and Big Five trait estimates.

#### 3.4.3. Network-Level Comparability (Structural–Functional Indices)

Criterion and structural–functional indices (e.g., global strength, network density) were used to compare the overall connectivity and parsimony of the DM and Big Five representational networks. DM and Big Five showed comparable global strength (DM = 12.4; B5 = 13.1) and similar network density (0.31 vs. 0.33), suggesting that the two systems yield networks of comparable complexity. These metrics describe the structure of the respective networks and do not by themselves imply strong person-level convergence.

Table 4 shows the pattern of cross-network associations of the convergent validity estimates. The table presents a summary of DM validity indices that compare the Big Five personality traits through both correlation-based and network-level metrics.

## 4. Discussion

### 4.1. Theoretical Contributions

The present work contributes to the emerging psychometrics of expressive–semantic assessment by evaluating Drawmetrics (DM) using complementary forms of validity evidence: (a) structural evidence about whether the semantic representation yields stable relational organization, and (b) score-level evidence about person-level linking to Big Five traits. The findings are most appropriately interpreted with respect to the measurement pipeline summarized in Figure 1, which separates expressive response generation from algorithmic score construction. In this framework, externally specified facet anchors define the semantic score space and open-response lexical descriptors are linked to that frame through similarity-based projection, yielding standardized person-by-facet composites. The reported reliability, indicator-level coherence, and network stability results therefore evaluate the behavior of these predefined composites and their relational organization, rather than post hoc discovery of new constructs. Taken together, the results support the feasibility of anchor-based semantic linking as a measurement strategy for open-response descriptors while keeping implementation details secondary to the linking structure itself. We combine pretrained sentence embeddings with network psychometric methods to evaluate internal structure, semantic-space alignment, and the extent to which DM can be interpreted in relation to conventional lexical trait models.

Across analyses, the most defensible claim is not that DM reproduces Big Five scores, but that DM yields a coherent expressive–semantic trait space that can be linked to Big Five constructs. Semantic-space alignment indices suggest that DM facet language is interpretable within the broad five-domain organization of lexical trait models, supporting the interpretation of DM facets in familiar terms. At the same time, person-level correlations between DM and Big Five domains were modest in the present cohort (mean |r| ≈ = 0.07; Spearman’s rho), indicating that DM is not interchangeable with the Big Five inventory and likely captures both overlapping and distinct variance.

DM domains showed strong relational structure stability under internal-consistency and hierarchical partitioning indices (ω and ω_h_), consistent with coherent within-domain organization of the indicator sets used in scoring. In addition, network-based resampling analyses suggested that key structural features (centrality ordering, edge patterns, and modular organization) were replicable under bootstrap perturbations. These results support the claim that DM yields a stable representational organization under the current scoring and modeling choices, even when person-level convergence with Big Five scores is modest.

The strongest evidence for overlap appears at the representational level (structural homology), where canonical and geometric similarity indices (e.g., canonical correlation, RV, Procrustes, distance correlation) indicate that the two systems share a nontrivial organizational pattern in facet space. Because both instruments’ facet descriptors are embedded in the same pretrained language model, high configuration-level alignment is expected and should be read as evidence for translation/linking of vocabularies rather than equivalence of respondent scores. These indices should be interpreted as evidence that the trait-language map learned by DM is compatible with the Big Five map and helpful for linking and translation, but not as evidence that the instruments yield equivalent person-level trait estimates. Future work will focus on improving score scaling (including addressing floor effects and zero inflation), quantifying temporal stability, and evaluating criterion outcomes to test whether DM offers incremental validity beyond lexical self-report.

### 4.2. Expressive Versus Lexical Models of Personality: Conceptual Integration

The Big Five personality model and other traditional frameworks originate from the lexical tradition, which posits that socially salient characteristics are encoded in natural language and can be identified through factor analysis of trait-descriptive adjectives (Goldberg, 1992; John et al., 1988). Unlike purely lexical self-report methods, Drawmetrics (DM) employs expressive production and associated descriptors to construct a semantic representation of attitudinal content. The findings indicate that DM can be interpreted within the Big Five lexical framework at the level of representational organization (structural homology). Yet, it produces person-level scores that are not interchangeable with Big Five trait levels in this sample. This supports a conceptual integration in which lexical models offer an established interpretive scaffold, while expressive–semantic models may capture trait-relevant information through distinct mechanisms of elicitation and encoding.

A key implication is that lexical self-descriptions may diverge from actual behavior and presentation, especially in contexts involving impression management or limited self-insight. DM operationalizes personality using vector-based embeddings that quantify attitudinal meaning from doodle-based representations, their descriptors, and related nonverbal or semantic outputs. In this study, DM demonstrated broad consistency with the five-domain lexical scaffold at the representational level. However, its current scoring scheme yielded only modest person-level covariation with Big Five domains, supporting a linked-but-not-identical interpretation. These findings support an assessment framework that integrates lexical and expressive evidence to examine personality through both self-description and expressive production. DM thus unites lexical traits with attitudinal meaning, establishing a psychometric framework that connects language, symbolism, and behavioral expressions in personality assessment.

### 4.3. Applied Contributions

The present work advances applied personality assessment by developing and evaluating DM as an expressive–semantic tool that can complement traditional self-report questionnaires. Because DM is based on expressive production and semantic scoring rather than direct Likert endorsement, it may be useful in contexts where translation burden, response styles, or impression management limit self-report. DM also supports automated, scalable scoring through NLP and network-analytic methods, enabling standardized processing in high-volume or remote administration settings.

The primary applied contribution of this work is interpretive: DM can be associated with established Big Five trait language at the representational level, which supports the communication of results using a recognized lexical framework. However, given that person-level DM–Big Five covariation was modest under the current scoring and sample conditions, DM should not be regarded as a substitute for a Big Five inventory. The most appropriate near-term application is as a complementary signal, such as in research on behavioral or attitudinal patterns, coaching and development, and criterion-validation studies, until stronger evidence emerges regarding predictive validity, temporal stability, and subgroup performance in applied contexts.

### 4.4. Limitations and Future Directions

The findings provide preliminary evidence that DM outputs can be linked with FFM facet language through an embedding-based mapping approach combined with expert audit. Analyses indicate that DM-derived composites exhibit interpretable organization and internal relationships that align with expected FFM facet and domain categories. However, these results should be interpreted as evidence of linking or alignment, given that the analytic representation was explicitly constructed to correspond to IPIP-NEO facets. Therefore, the study does not demonstrate that DM responses independently recover the FFM dimensional structure without imposed correspondences, nor does it establish the interchangeability of person-level scores. Furthermore, representational alignment is influenced by the choice of embedding model and descriptor set. Replication using alternative pretrained models (e.g., MPNet or larger transformer encoders), different facet label sets, and varied scoring normalizations is recommended.

## Figures and Tables

**Table 1:** Relational Structure Stability (Omega-Based Internal Consistency) for Drawmetrics Domains.

Domain	N	k	ω	95% CI (ω)	ω_h_	Interpretation
Agreeableness (A)	176	56	0.94	[0.94–0.95]	0.74	High reliability; substantial general-factor saturation (ω = 0.94; ω_h_ = 0.74)
Conscientiousness (C)	176	32	0.86	[0.84–0.89]	0.46	Good reliability; mixed general and domain-specific variance (moderate ω_h_)
Extraversion (E)	176	17	0.85	[0.83–0.88]	0.49	Good reliability; mixed general and domain-specific variance (ω_h_ = 0.49)
Openness (O)	176	9	0.71	[0.65–0.76]	0.65	Acceptable ω; strong general-factor saturation relative to total reliability (ω_h_ = 0.65), consistent with heterogeneous indicators
Neuroticism (N)	176	3	0.85	[0.81–0.88]	-	Reliable short composite; ω_h_ not reported due to k = 3 (unstable for hierarchical partitioning)

Note. *k* = number of Drawmetrics indicators (terms/features) included in each domain composite. ω = McDonald’s omega total; ω_h_ = omega hierarchical derived from a Schmid–Leiman decomposition of the indicator correlation matrix and reported here as evidence of hierarchical structure (general vs. domain-specific variance), not as a confirmatory SEM model fit. CI = 95% percentile bootstrap confidence interval (1000 resamples). ω_h_ is not reported for Neuroticism due to small *k* (3), for which hierarchical estimates are unstable. Estimates were computed from the [Pearson] indicator correlation matrix.

**Table 2:** Indicator–Total Correlation Diagnostics for Drawmetrics Domains.

Domain	N Indicators	Mean r_it_	Min r_it_	Max r_it_	Interpretation
Agreeableness	56	0.21	0.08	0.38	Moderate homogeneity; indicators vary in contribution
Conscientiousness	32	0.24	0.11	0.39	Moderate–strong coherence; consistent with good reliability
Extraversion	17	0.22	0.14	0.29	Moderate cohesion across indicators
Neuroticism	3	0.20	0.18	0.23	Short composite; items consistent but limited coverage
Openness	9	0.20	0.07	0.30	Heterogeneous item set; acceptable for expressive data

Note. r_it_ = corrected indicator–total correlation computed within each domain. Given semantically scored open-response indicators, variability in r_it_ is expected and is interpreted as differential indicator contribution rather than item malfunction. Indicator–total correlations are interpreted cautiously for very small indicator sets (e.g., k = 3).

**Table 3:** Network Stability Metrics for Drawmetrics and Big Five Networks.

Metric	Index	Drawmetrics	Big Five	Interpretation	Reference
Centrality Stability (CS)	Strength	0.73	0.78	Excellent (>0.70); highly stable central nodes	Epskamp et al. (2018)
Centrality Stability (CS)	Betweenness	0.58	0.61	Good (>0.50); moderately stable; interpret cautiously	Epskamp et al. (2018)
Centrality Stability (CS)	Closeness	0.55	0.63	Good (>0.50); moderately stable; interpret cautiously	Epskamp et al. (2018)
Edge Stability	Mean Retention	0.82	0.86	High reproducibility of edges across 1000 bootstraps	Epskamp et al. (2018)
Edge Stability	Median Edge r	0.88	0.91	Strong correspondence of edge weights	Epskamp et al. (2018)
Modularity (Louvain Q)	—	0.42	0.48	Consistent five-domain modularity structure	Epskamp et al. (2018)
Global Strength	Sum of Edge Weights	12.4	13.1	Comparable overall connectivity and parsimony	Epskamp et al. (2018)
Proportion of realized edges	Edges/(Nodes)	0.31	0.33	Balanced network sparsity; avoids overfitting	Epskamp et al. (2018)

Note. CS = centrality stability coefficient; Q = Louvain modularity index; Edge retention and correlation values were derived from 1000 bootstrap replications. Values above 0.50 indicate good stability, and values above 0.70 indicate excellent reliability of network features. Network density was calculated as the proportion of realized edges relative to all possible edges among terms, providing an estimate of global connectivity and model parsimony.

**Table 4:** Summary of Validity Metrics for Drawmetrics and Big Five Models.

Validity Type	Metric	Drawmetrics (DM)	Big Five (B5)	Interpretation	Reference
Score-level Linking	Domain *r* (matched domains, Pearson)	mean |r| = 0.067 (−0.083 to 0.122)	—	Modest person-level covariation; supports linking rather than equivalence	—
Score-level Linking	Domain ρ (matched domains, Spearman)	mean |ρ| ≈ 0.067 (−0.122 to 0.117)	—	Monotone association is also modest in this cohort	—
Score-level Linking	Max |r| (facet-level)	|r| = 0.259 (n = 133; Orderliness × Tends toward sadness)	—	Largest observed facet-level covariation (pairwise *N* varies)	—
Semantic-space Alignment	First canonical correlation (CCA; r_c1})	1.00	—	Reported descriptively; inference emphasized via permutation-based and distance-geometry indices	—
Semantic-space Alignment	RV coefficient	0.937	—	High overall configuration similarity (RV); interpret alongside permutation tests	Escoufier (1973)
Semantic-space Alignment	Procrustes similarity	0.94	—	Strong geometric correspondence after optimal rotation	Gower (1975)
Semantic-space Alignment	Distance correlation	0.968	—	Very strong dependence between configurations (including nonlinear)	Székely et al. (2007)
Semantic-space Alignment	Mantel *r* (cosine-distance matrices; permutation)	r = 0.385; *p* < 0.001 (5000 permutations)	—	Facet–facet relational geometry correspondence exceeds chance under label shuffling	—
Criterion/Network Structure	Global strength/density	Strength: 12.4; Density: 0.31	Strength: 13.1; Density: 0.33	Comparable overall connectivity and parsimony	—

Note. Mantel *r* evaluates global correspondence between pairwise distance matrices via permutation testing (Mantel, 1967). In the present context, it is used as a summary index of geometric alignment between semantic embedding spaces, rather than as a test of local structure or model equivalence. Consistent with prior methodological critiques (Legendre & Legendre, 2012), the statistic is interpreted here as a descriptive measure of overall geometric alignment and not as evidence of structural identity, metric invariance, or localized correspondence.

## Data Availability

The deidentified DM and Big Five datasets, as well as the analytic code used for this research, are proprietary to AttituX Ltd. Pte. and are not available for public access or external sharing. Results were derived from using internal analytic infrastructure in compliance with organizational data governance policies.

## Abbreviations

The following abbreviations are used in this manuscript:

DM	Drawmetrics
BERT	Bidirectional Encoder Representations from Transformers
BGGM	Bayesian Gaussian Graphical Model
BF	Big Five
BFI	IPIP-NEO-120
r(c)	canonical correlation
EGA	Exploratory Graph Analysis
NLP	Natural Language Processing
TGA	Taxonomic Graph Analysis
$\bar{r}$	discriminant separations expressed as an average correlation
ω	coefficient Omega
ω_h_	coefficient Omega hierarchical
CS	coefficient of stability in network psychometrics
Q	Louvain modularity index
r	correlation coefficient
